# Antiepileptic drugs and bone metabolism

**DOI:** 10.1186/1743-7075-3-36

**Published:** 2006-09-06

**Authors:** Helen A Valsamis, Surender K Arora, Barbara Labban, Samy I McFarlane

**Affiliations:** 1 Department of Neurology, SUNY Downstate Medical Center, and Kings County Hospital Center, Brooklyn, NY 11203, USA; 2 Division of Endocrinology, Diabetes and Hypertension, SUNY Downstate Medical Center, and Kings County Hospital Center, Brooklyn, NY 11203, USA; 3 Department of Medicine, Staten Island University Hospital, NY 10305, USA

## Abstract

Anti-epileptic medications encompass a wide range of drugs including anticonvulsants, benzodiazepines, enzyme inducers or inhibitors, with a variety effects, including induction of cytochrome P450 and other enzyme, which may lead to catabolism of vitamin D and hypocalcemia and other effects that may significantly effect the risk for low bone mass and fractures. With the current estimates of 50 million people worldwide with epilepsy together with the rapid increase in utilization of these medications for other indications, bone disease associated with the use of anti-epileptic medications is emerging as a serious health threat for millions of people. Nevertheless, it usually goes unrecognized and untreated. In this review we discuss the pathophysiologic mechanisms of bone disease associated with anti-epileptic use, including effect of anti-epileptic agents on bone turnover and fracture risk, highlighting various strategies for prevention of bone loss and associated fractures a rapidly increasing vulnerable population.

## Background

Epilepsy is a major public health problem affecting nearly 50 million people worldwide [1]. Treatment with anti epileptic drugs (AEDs) is generally chronic, if not life long and may be associated with significant metabolic effects including decreased bone mass and increased fractures [2,3]. AEDs include a variety of drugs that may lead to catabolism of vitamin D and hypocalcemia. In addition, many of these medications such as the benzodiazepines, carbamazepine (CBZ) and clonazepam (CZP), and barbiturates, are currently utilized in disorders other than epilepsy such as pain and mood disorders and the problem may be a general one [4].

The adverse effects of AEDs on bone health were first reported nearly four decades ago [5,6] and since then a mounting body of evidence has linked a variety of biochemical, metabolic and radiologic abnormalities in bones to the use of AEDs [2,3,7]. AEDs have been identified as an independent risk factor for low bone density and osteoporosis [8].

Low bone mass associated with AED use is largely unrecognized, undetected, and untreated [2,3]. In a survey of 624 adult and pediatric neurologists designed to assess the awareness of the effects of AED therapy on bone health, among treating physicians, only 28% of adult and 41 % of pediatric neurologists reported screening their patients for bone disease. In this cohort of neurologist, only 7% of adult and 9% of pediatric neurologists prescribed prophylactic calcium and vitamin D for patients receiving AED treatment [9]. These data underscore the need for aggressive educational strategies to increase screening and treatment of metabolic bone disorders associated with AED use by the treating physicians. This article will examine the effects of AEDs on bone health in persons with epilepsy in light of the current understanding of the mechanisms of bone disease. We will also discuss the possible preventive and therapeutic options for bone loss in this patient population.

### Bone structure and metabolism

**Figure 1:**
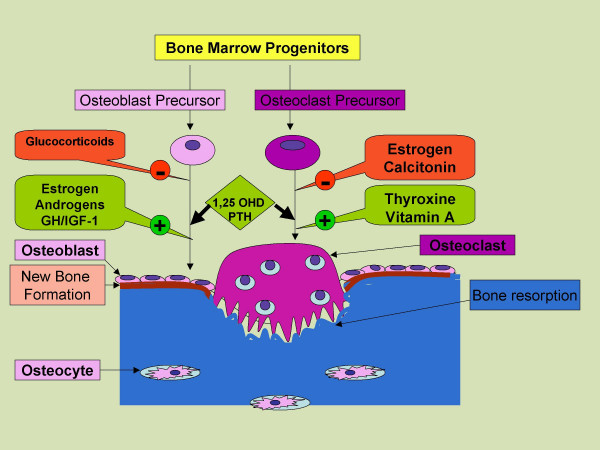
**Development of osteoblasts and osteoclasts from bone marrow progenitors**. Factors affecting the development and function of these cells, bone resorption by osteoclast and new bone formation by osteoblasts. **Abbreviations: GH**: growth hormone, **IGF**: insulin like growth factors, **PTH**: parathyroid hormone

Bone is a dynamic tissue that is remodeled continuously throughout life (Figure 1). Specialized cells called osteoblasts initiate bone formation, osteocytes monitor bone mechanical stresses, and osteoclasts resorb bone. Bone density is determined by the dynamic balance between bone formation and bone resorption. Formation starts with the deposition of an organic matrix by osteoblasts followed by the process of mineralization [10]. The organic matrix is comprised predominantly of type I collagen (90–95%) with contributions from various other proteins including osteocalcin, osteonectin, osteopontin and thrombospondin [10]. The mineral phase of bone, hydroxyapatite, is composed of calcium and phosphorus. The concentration of these ions in plasma and extracellular fluid determines the rate at which hydroxyapatite is formed and deposited. There is a critical limit for the concentrations of calcium and phosphorus ions below which the mineralization of organic matrix does not occur [10].

The functions of osteoblasts are controlled by a variety of growth factors including insulin like growth factors I and II, parathyroid hormone (PTH), and vitamin D_3 _[1, 25 (OH) _2 _D_3_] [10]. Increased osteoblast activity results in elevated serum concentration of bone-specific alkaline phosphatase and osteocalcin which serve as serum markers of bone formation [10]. Histologically, active osteoblasts are distinguished by a specific skeletal form of alkaline phosphatase, and receptors for PTH and vitamin D_3 _[10].

After the deposition and mineralization of the bony matrix, the osteoblasts transform into osteocytes and function as mechanosensors. They sense mechanical stresses and transmit signals to initiate bone remodeling along the lines of force produced by these stresses [10]. Remodeling requires recruitment of osteoclasts, multinucleate cells responsible for bone resorption. Osteoclasts are indirectly regulated by osteoblasts although their differentiation and function is also modulated by a number of growth factors and cytokines including tumor necrosis factor, interferon gamma, and interleukins. Osteoclasts have a variety of lysosomal enzymes including proteinases, a specialized proton pump ATPase, and carbonic anhydrase type II. These enzymes create and maintain an acid environment to solubilize the mineral phase and resorb the bone matrix [10]. This is followed by well coordinated osteoblastic activity to lay down new bone in the areas of bone resorption and restore the bone strength along the lines of stress (Figure 1).

A number of biochemical markers can be measured that reflect the overall rate of bone remodeling. These can be divided into markers of bone formation derived from osteoblasts and markers of bone resorption representing degraded products of osteoclastic activity [10]. Alkaline phosphatase is a marker for osteoblasts and its cellular levels correlate with rates of bone formation. Other circulating markers of bone formation include osteocalcin and type I procollagen C-terminal peptide (PICP) [10]. Urinary markers of bone resorption include hydroxyproline, hydroxylysine and bone specific hydroxypyridinium collagen cross links. Bone turn over and hence bone markers are physiologically elevated during growth periods and bone repairs [10]. In healthy individuals, bone turnover is a function of age and levels of bone markers reflect the state of bone remodeling at any given point of time. Their primary use is for monitoring excessive bone remodeling and the response to treatment of osteoporosis [10].

Bone remodeling is regulated by several circulating hormones and growth factors including estrogens, androgens, vitamin D, PTH, Tumor necrosis factor (TNF) and insulin like growth factors (IGF) I & II. Nutrition, calcium intake and physical activity also influence bone remodeling. Increased sex hormone production at puberty is crucial for achievement of peak bone mass. Bone loss may be exaggerated in women after menopause or pathologically, in either sex, with any form of hypogonadism [10]. Many other factors, including medications such as steroid & AEDs, have been implicated for low bone density and increased risk of fractures.

Both PTH and vitamin D play important roles in development and maintenance of bone mass by maintaining calcium and phosphate homeostasis and modulating osteoblastic and osteoclastic functions. PTH increases osteoclastic bone resorption by activating the receptor activator of NFkappaB ligand (RANKL) on osteoblasts. Vitamin D_3 _promotes differentiation along the osteoclastic pathway through receptors located on osteoclast precursors [10]. Elevated PTH levels, either as primary abnormality or as a compensatory response to hypocalcemia, can activate osteoclastic bone resorption to maintain normocalcemia. Deficiency of vitamin D may adversely affect mineralization of bone matrix and compromise bone strength and is an established independent risk factor for low bone mass and fracture [11,12]. As dietary sources of vitamin D are limited, most people depend on adequate sun exposure to ensure cutaneous synthesis of vitamin D from cholesterol precursors. This is particularly important in geographic areas with limited sunlight such as the northern hemisphere [13]. Severe vitamin D deficiency results in defective mineralization of the skeleton predisposing to rickets in children and osteomalacia in adults [11].

Bone mineral density is a surrogate marker for measuring bone mass and bone strength. In a healthy population, skeletal mass and BMD increases throughout childhood and adolescence with achievement of peak bone mass in early adulthood [10]. After the age of 30–45 years, bone resorption exceeds bone formation and BMD begins to decrease. This imbalance may begin at different ages and may be variable at different skeletal sites [10].

**Figure 2:**
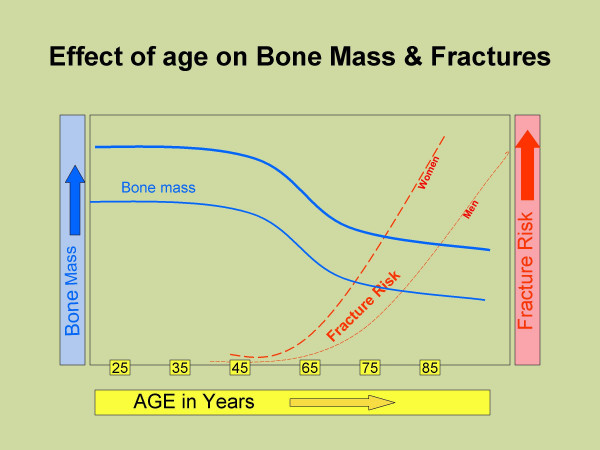
Decrease in peak bone mass with age and increase in fracture risk with increasing age.

The gold standard for BMD measurement is Dual-Energy X-ray Absorptiometry (DEXA) with an accuracy of up to 99% at any given site [14]. The measurement is usually made at the lumbar spine, femoral neck, and forearm providing representative samples of trabecular, mixed, and cortical bone respectively [14]. Results are expressed as a T score, the number of standard deviations (SD) from the mean peak BMD for a given population. Osteoporosis is defined clinically as the presence both of a fragility fracture and of low bone mass. Operationally, osteoporosis is defined as a bone density more than 2.5 SD below the mean peak BMD (T score < -2.5) and osteopenia is defined as BMD between 1 and 2.5 SD below the mean peak value (T score <-1 & > -2.5) while BMD with T score > -1 is reported normal. The fracture risk correlates well with the bone mass & BMD and increases 2 fold with each SD decrease in BMD [14] (Figure 2).

### Effects of epilepsy on bone

Epilepsy increases the risk for fracture by a variety of mechanisms in addition to those attributed to the use of AEDs. The fracture rate in patients with epilepsy is 2–6 times higher than the rate observed in the general population [15-17]. This increase in fracture risk in subjects with epilepsy is comparable to that seen with chronic steroid use [18] and represents an increased risk of falls as well as increased bone fragility.

Falls are more common in patients with epilepsy as compared to matched controls [16-18]. Interestingly, seizure activity does not explain the majority of falls in epileptic patients; approximately two thirds of falls occur in the absence of a seizure [19] with only one third directly attributed to seizure activity [20,21]. Epilepsy may be part of a syndrome associated with a variety of neurological deficits leading to weakness, loss of coordination, altered sensory modalities and impaired cognition. Furthermore, most of the AEDs, benzodiazepines, anticonvulsants such as valproic acid and barbiturates, have CNS effects, and their use is associated with neurologic side effects including somnolence, ataxia and tremor which may also contribute to gait disturbances with consequent increased risk of falls and fractures [22]. In a prospective cohort study of more than 8,000 community-dwelling women older than 65 years participating in the fourth examination of the Study of Osteoporotic Fractures, AED users were 75% more likely to have a fall compared to those who did not use AEDs [23]. In this study, other CNS-active medications such as benzodiazepines and antidepressants were associated with increased risk of falls. These findings underscore the importance of the CNS-related effects of AEDs in increasing fracture rates.

Quality of life studies in patients with epilepsy suggest that activity is frequently limited by the patients and their families out of concern for provoking a seizure and its consequences [24]. Immobility, inactivity, and fewer weight bearing activities are strong risk factors for osteoporosis [10]. Additionally, many persons with epilepsy are institutionalized which may decrease their activity status. Institutionalized patients have high rates of neurologic comorbidities, further decreasing mobility. The presence of focal weakness and immobility may induce osteopenia and bone loss in the affected limb or limbs. This may even occur in the axial skeleton if immobility is prolonged or if subject is bed ridden [10]. Studies in institutionalized patients with epilepsy have demonstrated high risk of fracture in this population [25].

A number of earlier studies reported rickets and osteomalacia in institutionalized subjects with epilepsy [6,26,27]. Recent studies did not observe significant presence of rickets or osteomalacia in patients with epilepsy who are ambulatory [28-30]. However, milder vitamin D deficiency is fairly common in this population and has been reported in more than half of medical inpatients [13]. More subtle deficiency of vitamin D may be associated with secondary hyperparathyroidism and increased bone turn over and may predispose to rapid bone loss and osteoporosis. Patients with epilepsy who are housebound or institutionalized patients with epilepsy are at high risk of vitamin D deficiency due to inadequate sunlight exposure. In addition, sun exposure is often restricted with AEDs such as carbamazepine, to reduce the occurrence of sun light induced rash.

## Effects of AEDs on bone metabolism

### Effect on fracture rates

Accumulating evidence has linked AED use with increased fracture rates in both the community-dwelling [31] and hospitalized populations [25]. In a population based study, the use of AEDs was associated with significantly high risk of fracture (odds ratio 1.14–1.79) and a dose response relationship was reported with carbamazepine (CBZ), phenobarbital (PB), oxcarbazepine (OXC), clonazepam (CZP) and valproate (VPA). Fracture risk was higher for hepatic enzyme inducing AEDs such as phenytoin (DPH), CBZ and PB, than for non-inducing AEDs [21]. In another large population based study, continued AED use was associated with higher risk of bone loss at hip sufficient to increase the risk of hip fracture by 29% over 5 years [22].

### Factors affecting bone remodeling

Since the first reports of adverse bone effects of AEDs more than 3 decades ago [5,6,26], a number of biochemical abnormalities of bone metabolism have been reported with AED use including hypocalcemia, hypophosphatemia, low vitamin D levels and increase in PTH [2,3,7,32]. This constellation of effects has been demonstrated in both children and adults and is commonly seen with AEDs that induce cytochrome P450 enzymes, particularly DPH, CBZ and PB [33-38]. These enzyme inducing AEDs may increase catabolism of vitamin D resulting in hypophosphatemia and hypocalcemia. Moreover, AEDs may inhibit cellular response to PTH. Both mechanisms have been proposed and may contribute to increases bone remodeling [3,39]. DPH has additional effects that can lead to hypocalcemia. DPH decreases intestinal cation transport and calcium absorption as well as vitamin D mediated calcium absorption[3,7]. However, the data are not consistent and many studies failed to observe any significant decrease in serum calcium and phosphate levels with use of enzyme-inducing AEDs [30,40,41].

VPA, a hepatic enzyme inhibitor, is also associated with decreased BMD and is thought to act by stimulating osteoclast activity [28]. Studies of VPA and calcium levels are contradictory [42,43].

Decreased urinary excretion of calcium with use of AEDs (CBZ, DPH, VPA) has been reported in both children [34] and adults [33]. Surprisingly, in the latter study [33], decrease in urinary calcium excretion with CBZ and DPH was limited to female participants only. The mechanism underlying this hypocalciuric effect of AEDs is unknown and needs further exploration. Although the evidence for newer AEDs is still limited, lamotrigine and topiramate have not been shown to cause significant effects on serum calcium and phosphate[2,3,7].

Low levels of biologically active vitamin D in patients on AEDs have been demonstrated in a number of studies [33,37,44-46], particularly with use of hepatic enzyme inducing medications like PB, PD, DPH and CBZ. This effect has been attributed to metabolism of vitamin D to polar inactive metabolites by the hepatic microsomes [3]. However, the data are not consistent and some studies have not shown significant reductions in vitamin D levels with use of AEDs[30,47].

Elevation of serum parathyroid hormone levels has been reported with use of AEDs in subjects with epilepsy [33,36]. This rise in PTH levels likely represents a secondary response to low vitamin D levels. However, high PTH has also been demonstrated independent of vitamin D deficiency [36]. High PTH levels may increase bone turnover and predispose to low bone mass [36]. High bone turnover has also been demonstrated with AED use despite normal levels of PTH [42,44]. Another proposed mechanism for the bone effects of AEDs is the inhibition of the cellular response to PTH [3]. Animal studies have shown that use of PB and DPH was associated with impaired PTH response [3] although this effect has not been confirmed in human studies.

### AEDs and markers of bone turnover

Increase in the markers of bone remodeling with use of AEDs has been demonstrated in a number of small studies [42,46-51]. Increases in serum levels of total and bone specific alkaline phosphatase, osteocalcin and PICP have been reported, often associated with increase in bone resorption markers such as N-telopeptide of type I collagen (NTX) and carboxy-terminal telopeptide of type I collagen (ICTP) [42,46-51]. These markers may be increased even in the presence of normal vitamin D and PTH levels [47]. Increased bone turnover has been observed in both children and adults and has been associated with use of enzyme inducing AEDs, particularly DPH, CBZ and the enzyme inhibitor, VPA. In many cases, the increased bone turnover was associated with decrease in BMD and may be an important contributing mechanism [42,44,51]. Histologically, biopsies of patients treated both with enzyme-inducing and enzyme-inhibiting AEDs suggest the bone disease is due mainly to an increased frequency of remodeling activation and bone turnover, rather than a mineralization defect [52]. Data from the newer, non-enzyme inducing AEDs are still limited.

Gender may influence the response of bone to AEDs as some studies have shown different responses in men and women. One study reported decreases in urinary deoxypyridinoline levels in male epileptic patients receiving multiple AEDs but not in women or with use of single AED [33].

### Effects of AEDs on bone density

Many epidemiological studies now link AED use to decreased bone mass in both sexes [22,29,53]. In a large study of postmenopausal Caucasian women 65 years and older [22], continued use of AEDs was associated with almost 2 fold increase in rates of bone loss at hip and calcaneous, sufficient to increase the risk of hip fracture by 29% over 5 years. DPH was the only AED where a specific significance could be assigned. The other AEDs were used in insufficient numbers of patients. In one study of 81 men in the age group of 25–54 years [53], significant declines in femoral neck BMD (annualized loss 1.8%) were observed in those in the youngest age group (25–44 years), suggesting that effect of AED may be more pronounced in the younger rather than older age group. This is a particularly concerning as AEDs are typically used for long periods of time and may be used for life. No causal association was found with a specific type of AED. Age and duration of AED use correlated significantly with low femoral BMD [53]. Similar associations were also reported in children where significant decreases in height to less than 10^th ^percentile in children were seen with AED use [29].

Gender differences have been noted in the effects of AEDs on bone density. In several of the studies showing loss of bone mass with AED use, the effects are more marked in women [33,54] and in patients with restricted activity [29,40]. Duration of AED use also predicted bone loss as did polypharmacy[44,51,55,56], although small sample size and differing methodologies make these studies difficult to compare.

Hepatic enzyme inducing AEDs are implicated in bone loss as is the hepatic enzyme inhibitor, VPA. Of the newer AEDs, with fewer hepatic effects, LTG has been used in some studies without significant effect on bone metabolism [43]. The carbonic anhydrase inhibitors, topiramate and zonisamide, were linked to increased risk of fractures in a few small studies [7].

## Mechanism of bone loss with AED use

The exact mechanisms for adverse bone effects of AEDs have not been determined although many factors are known to influence and modify these effects (Table 1).

**Table 1:** Proposed mechanisms contributing to AED induced bone disease.

• Vitamin D Inactivation
• Hepatic enzyme induction
• PXR activation
• Altered calcium metabolism (DPH)
• ↓ Intestinal absorption
• ↓ vitamin D mediated absorption
• ↓ intestinal cation transport
• ↑ PTH
• Vitamin D insufficiency
• ↓ cellular response to PTH
• Vitamin K deficiency
• ↓ Calcitonin
• Osteoblast Inhibition
• Altered sex-steroid & SHBG metabolism
• Possible modulation of Aromatase activity

**Abbreviations: PXR**: Pregnane X receptor, **DPH**: phentyoin, **PTH: **parathyroid hormone, **SHBG**: sex hormone binding globulin

**Figure 3:**
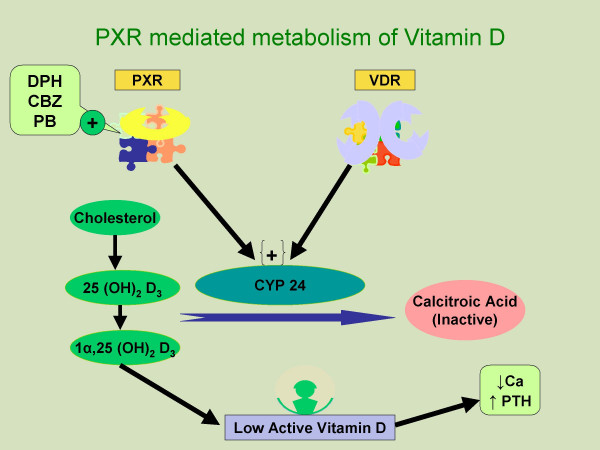
**Representation of pregnane X receptor (PXR) mediated vitamin D catabolism**. PXR is activated by various antiepileptic medications and other pharmaceutical agents and induces CYP 24, the enzyme which metabolises active vitamin D_3 _to inactive form. **Abbreviations: PXR**: Pregnane X receptor, **DPH**: phentyoin, **CBZ**: carbamazepine, **PB**: phenobarbital, **VDR: **vitamin D receptor, **PTH: **parathyroid hormone, **CYP 24**: 24-hydroxylase.

Enzyme-inducing AEDs such as DPH and CBZ have been shown to decrease vitamin D levels. Deficiency of vitamin D can lead to bone loss by causing hypocalcemia, hypophosphatemia and secondary hyperparathyroidism [11]. Low levels of vitamin D with AED use have been reported in many studies but significant correlation with BMD has generally not been observed [33,44,45]. Vitamin D has significant effects other than calcium homeostasis and secondary hyperparathyroidism. It is an important modulator of osteoblastic function and also facilitates differentiation along the osteoclastic lines [10]. Vitamin D deficiency associated with use of AEDs is likely mediated through the orphan nuclear receptor, pregnane X receptor (PXR) [57] (Figure 3). The PXR shares 60% homology in their DNA binding domains with the vitamin D receptor (VDRs) and is expressed in intestine, kidney and liver. PXR has been shown to mediate induction of CYP 2 and CYP 3, the cytochrome P450 enzymes involved in the drug metabolism. Furthermore, PXR can be activated by a variety of pharmaceutical agents including phenytoin, phenobarbital, carbamazepine and rifampicin [57]. Emerging evidence shows that these PXR activators can increase the expression of the CYP24, a VDR target gene in cultured cells and *in vivo *in mice. CYP 24 is an enzyme that directs the side chain oxidation and cleavage of 25 (OH)_2 _D_3 _and 1β, 25 (OH)_2 _D_3 _to carboxylic acid end products (calcitroic acid), resulting in lower cellular concentration of active vitamin D. This induces a state of vitamin D deficiency and results in hypocalcemia, secondary hyperparathyroidism and increased bone turnover predisposing to low bone density and bone loss [57,58]. This, however, does not explain the deficiency of vitamin D with VPA reported in some studies as VPA is an inhibitor of cytochrome P450 enzymes and is not among the known activators of PXR.

With the demonstration of significant homology between VDR and PXR, it is conceivable that the effects of PXR activation by pharmaceutical agents like AEDs may be not be limited to inactivation of vitamin D and may potentially interfere with many other VDR controlled physiological processes including cellular and bone effects [57,58]. These may affect growth and maturation of osteoclasts and function of osteoblasts leading to adverse effects on bone health [57].

Decreased vitamin D_3 _levels may affect osteoblast activity through the aromatase pathway [59]. Aromatase is an enzyme present in many extragonadal tissues including bones, liver, skin and functions to convert the circulating androgens to estrogens [59]. Evidence from a study in postmenopausal women suggests positive correlation between BMD and serum dehydroepiandrosterone sulfate (DHEAS) and estrone levels but not with estradiol levels [59,60]. This suggests that conversion of adrenal androgens to estrogen in peripheral tissues plays an important role in maintaining BMD in postmenopausal women. Vitamin D_3 _is an important regulator of aromatase activity and physiological concentrations of vitamin D_3 _are necessary for maintenance of aromatase activity in osteoblasts [59]. In addition, AEDs can decrease the availability of androgen substrates for aromatase pathway by increasing the catabolism of sex steroids and increasing SHBG levels, resulting in reduced free testosterone and DHEA levels [61-64].

Many studies have shown AEDs increase bone turnover and this may contribute to bone loss[3,7]. Factors associated with AED use, such as vitamin D deficiency, hyperparathyroidism and calcitonin deficiency [50], have been implicated in causing increased bone turnover. DPH and CBZ have been demonstrated to inhibit the proliferation of human osteoblast-like cell at concentrations equal to therapeutic doses[44]. VPA, a hepatic enzyme inhibitor, has been reported to act by stimulating osteoclast activity [28] and may cause imbalance between bone formation and resorption, contributing to bone loss. DPH has also been shown to inhibit osteocalcin secretion from osteoblasts [65] and may have more than one mechanism for bone effects.

DPH can cause vitamin K deficiency by increasing its metabolism and is frequently associated with hemorrhagic disease of the newborn when used in pregnant woman. Vitamin K is an essential cofactor for post translational carboxylation of various Gla bone proteins including osteocalcin [7]. DPH induced vitamin K deficiency can potentially cause bone loss by preventing the post translational modification of the vitamin K dependent matrix proteins. Supplementation with vitamin K has been shown to reduce bone loss in DPH treated rats [3] though there are no data from human studies.

Hepatic enzyme inducing AEDs can alter the synthesis and metabolism of sex steroids, serum levels of sex hormone binding globulin (SHBG) as well as central feedback mechanisms [61,62,66]. CBZ, DPH and VPA have been reported to directly inhibit testosterone biosynthesis by Leydig cells of testes in animal studies[63]. Increased clearance of androgens, including testosterone has been reported with DPH, CBZ and PB [62]. Furthermore, chronic CBZ and DPH treatment can increase SHBG levels with resultant decrease in free testosterone, estradiol and androsteindione levels. These changes may predispose to accelerated bone loss in subjects taking AEDs by reducing the androgen substrates for aromatase activity [62,64].

### Management of AED-induced bone loss

In absence of long-term randomized controlled trials evaluating different therapeutic options, most of the recommendations for management of AED-induced bone loss must be considered empirical though consistent with current evidence.

#### Good bone health practices

Discussion of the risks associated with AEDs and good bone health practices should be part of the evaluation of a seizure patient. Good bone health practices include regular weight-bearing exercise, adequate sunlight exposure, adequate intake of calcium and avoidance of risk factors for osteoporosis such as smoking and alcohol use. High risk patients should be identified before start of AED treatment and evaluated as recommended in later section. High risk patients include institutionalized and non-ambulatory subjects, those with poor dietary habits and limited sun exposure such as at higher latitudes, those on multiple AEDs and with increased duration of AED use. Those with multiple traditional osteoporosis risk factors as well as low calcium and vitamin D levels should also be considered at high risk and treated aggressively.

### Calcium supplementation

Calcium supplementation with doses of 1–1.5 gm/day should be offered to all persons using AEDs, particularly in presence of multiple risk factors or documented low BMD [32,67].

### Vitamin D

Prophylaxis with vitamin D has been recommended for all subjects using AEDs [3,7,52,68,69]. Due to increased catabolism of vitamin D, higher than normally recommended doses (up to 4000 IU per day) of vitamin D may be required for optimal effect, particularly for those with low vitamin D levels, high risk of bone disease and/or with documented low BMD [11,52,68]. Since the current RDI of 400 IU of vitamin D is not considered sufficient even in healthy adults [11], a dose of 800–1000 IU/day of vitamin D is reasonable as a preventive therapy in subjects using AEDs. For those with documented vitamin D deficiency, treatment with 50,000 IU/week for 8 weeks has been recommended and can be repeated if vit D levels remain low after initial treatment. This may be followed by supplementation with vitamin D 50,000 IU once a month to maintain the levels above the threshold of insufficiency [11].

### Surveillance

There is no clear consensus on recommendations for surveillance of bone disease associated with AEDs. Based on our experience, we recommend the following:

a) Baseline and then 6–12 months monitoring of serum calcium, phosphate, alkaline phosphatase, PTH and vitamin D levels.

b) Baseline screening with DEXA scan before initiating AED treatment in all high risk adults such as post menopausal women or subjects with multiple risk factors and then periodically at 1–2 year intervals.

c) Subjects with intermediate risk such as those with epilepsy on AEDs and one other risk factor may be screened with DEXA scan after 2 years of AED treatment.

d) In the subjects with no other osteoporosis risk factors, screening with DEXA scan may be appropriate after 5 years on AEDs [32].

e) Subjects with abnormal levels of above parameters and those with multiple risk factors for osteoporosis or low BMD on DEXA scan should preferably be evaluated and managed by endocrinologists.

f) In children, the value of BMD measurement before the development of peak bone mass without establishment of adequate reference ranges is questionable and needs to be evaluated further. However, as various studies have detected low bone mass with DEXA in pediatric age group as well, it should be probably offered to those at high risk as mentioned previously.

### Treatment

Recommendations for treatment of AED associated low bone mass depend on the severity of the bone disease.

a) Subjects with normal bone mass (**T score > -1**) should be encouraged to follow good bone health practices in addition to calcium and vitamin D supplementation.

b) For subjects with osteopenia (**T score < -1 and > -2.5**), in addition risk modification as above, antiresorptive treatment may be indicated for those with significant disease (T score < -1.5) and multiple risk factors for low bone mass. These subjects may benefit from a specialist evaluation.

c) For adults with osteoporosis (**T score < -2.5**) and or fragility fractures, treatment with antiresorptive medications is indicated in addition to calcium & vitamin D supplementation. These subjects should preferably be evaluated by endocrinologist and secondary causes for low bone mass ruled out as appropriate.

d) In postmenopausal women, hormone replacement therapy may retard the bone loss but the possibility of increase in seizure activity needs to be seriously considered in addition to risk of thromboembolism and breast cancer.

e) Oral bisphosphonates (alendronate, risedronate and ibandronate) may be considered for adults with significant osteopenia or osteoporosis although there are no established data for its use in AED induced bone loss specifically. Vitamin D insufficiency should be treated prior to starting bisphosphonates. Newer bisphosphonates are now available which can be used once a month (Ibandronate). For subjects who cannot tolerate oral bisphosphonates, treatment with parenteral agents such as ibandronate may be considered.

f) BMD with DEXA scan should be monitored at regular intervals of 12–18 months to monitor the response to therapy. Bone resorption markers such as N-telopeptide (NTX) may also be used to monitor the response to therapy as an adjunct to BMD testing.

g) Role of bisphosphonates in children or adolescents with low bone mass prior to achievement of peak bone mass has not been evaluated. Such therapy should be offered only after consideration of potential risks and benefits and should be appropriately individualized.

## Conclusion

Although epilepsy represents a group of heterogeneous disorders and the AEDs have multiple pharmacologic effects, accumulating evidence shows that exposure to AEDs confers an increased risk of osteoporosis and fracture. The current evidence also suggests that young adults may be at particularly increased risk of bone loss. Low calcium intake and vitamin D deficiency may aggravate these bone effects and are potentially treatable factors. Inactivity is another important factor associated with bone loss and needs to be considered in the management of bone disease associated with AEDs. Despite the evidence suggesting adverse effects of AEDs on bone, there appears to be a general lack of awareness among physicians about these effects. Although there is a lack of any definitive screening or treatment guidelines, those screening, monitoring and treatments that are easily available are not effectively utilized. Since these bone effects of AEDs, particularly osteopenia and osteoporosis, tend to be serious and encompass significant health care and financial costs, more attention needs to given and guidelines for their screening and treatment need to established.

## Competing interests

The author(s) declare that they have no competing interests.

## Authors' contributions

HV conceived the idea & helped with preparation and organization of the manuscript, SKA researched the topic, prepared & organized the manuscript, BL helped with research & data collection, SIM conceived, organized, reviewed and contributed to the final presentation of the manuscript.
